# Supplementary figures and images for: A multi-omics analysis of human fibroblasts overexpressing an Alu transposon reveals widespread disruptions in aging-associated pathways
Source: GeroScience. 2025 Dec 11;48(3):3375–402. doi: 10.1007/s11357-025-02033-6 (PMC13356197; doi:10.1007/s11357-025-02033-6)

Fig. S1

**a** AluSc5 subfamily expression across age

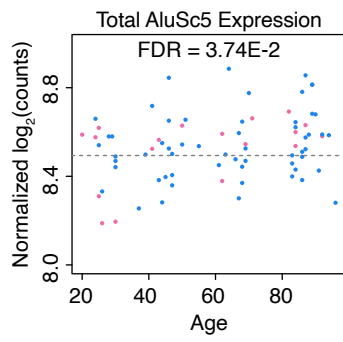

**b** AluYk11 subfamily expression across age

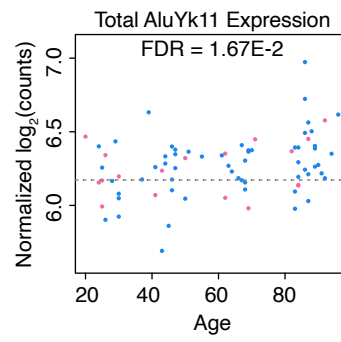

Supplement: Supplementary file 1 — Supplementary Fig. S1 Aging upregulates diverse Alu subfamilies in primary human fibroblasts. Scatterplots for the library size-normalized counts across age for (a) AluSc5 and (b) AluYk11 expression. Pink = Female, Blue = Male (PDF 29.3 KB) [file 11357_2025_2033_MOESM1_ESM.pdf]

Fig. S2

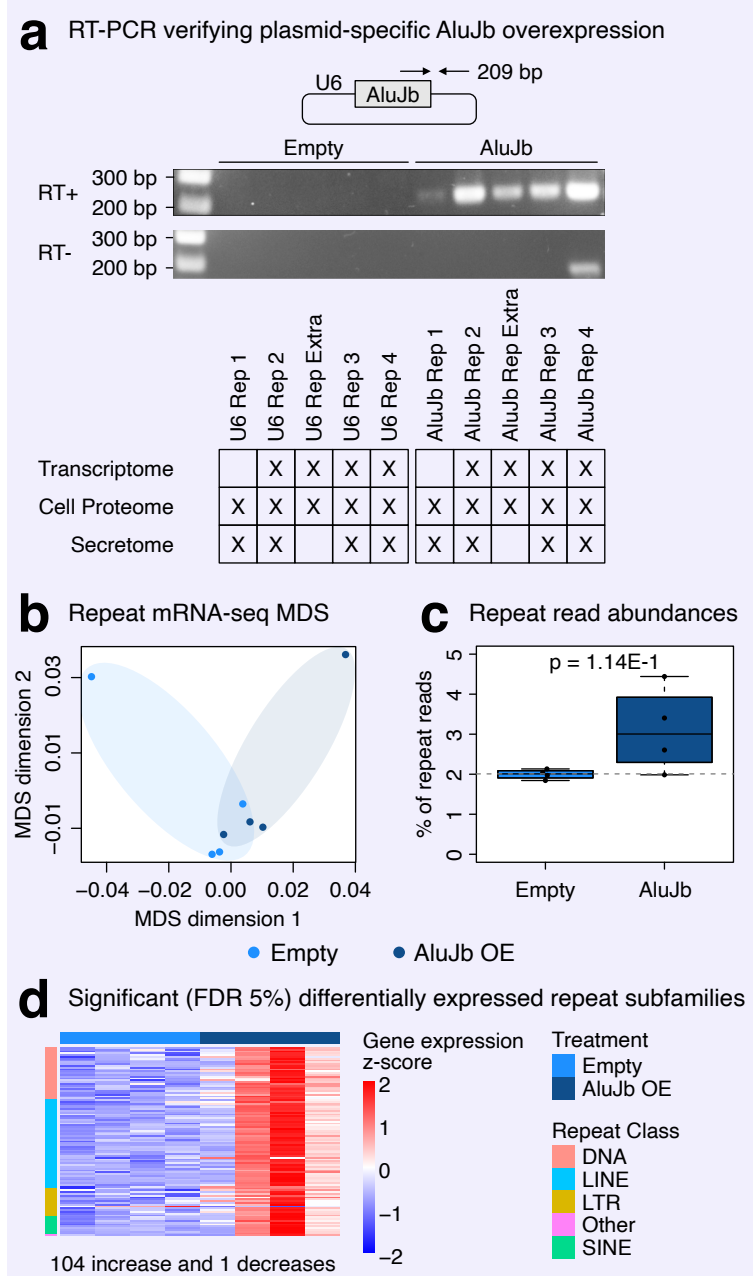

Supplement: Supplementary file 2 — Supplementary Fig. S2 Endogenous repetitive elements are upregulated following AluJb overexpression. (a) Plasmid-specific AluJb overexpression was assessed by endpoint RT-PCR of empty vector and AluJb overexpressing IMR-90 fibroblasts using primers targeting the 3’ AluJb-plasmid backbone junction. PCR reactions were carried out with (RT+) or without (RT-) reverse transcription in N = 5 samples per group, and N = 4-5 of these were used for multi-omic profiling. We note that the band on the lower right corner of the gel appears to be smaller in size than the expected amplicon size and may correspond to primer dimers. A table showing samples utilized for each -omics analysis is also shown. (b) Multidimensional scaling (MDS) analysis of the repetitive element transcriptome across samples. (c) Quantification of the percent of total reads mapping to repetitive elements. Statistical significance was assessed with a Wilcoxon rank sum test. (d) A gene expression heatmap for significant (FDR < 0.05) differentially expressed repeat subfamilies. RT: Reverse Transcription, FDR: False Discovery Rate (PDF 102 KB) [file 11357_2025_2033_MOESM2_ESM.pdf]

Fig. S7

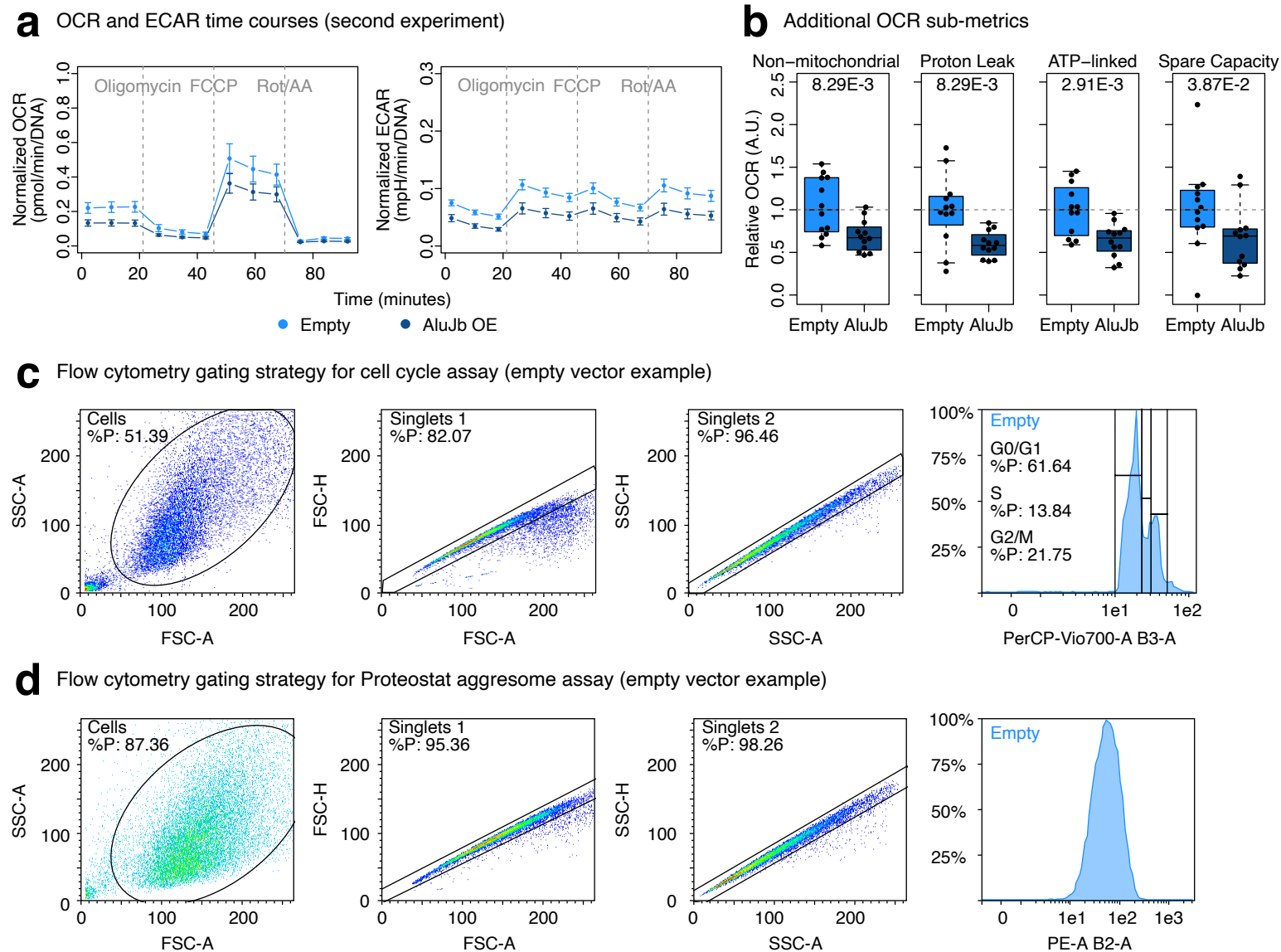

Supplement: Supplementary file 7 — Supplementary Fig. S7 Additional functional profiling data and parameters. (a) OCR and ECAR time courses from a second experiment with N = 6 independent transfections per group, for a total of N = 12 samples per group. (b) Additional OCR sub-metrics, including non-mitochondrial respiration, ATP-linked respiration, proton leak, and spare capacity, in empty control and AluJb-overexpressing IMR-90 fibroblasts (N = 12 per group). Statistical significance was assessed with a Wilcoxon rank sum test, and p < 0.05 was considered significant. The flow cytometry gating strategies for the (c) propidium iodide-based cell cycle assay and the (d) Proteostat aggresome detection assay. OCR: Oxygen Consumption Rate, ECAR: Extracellular Acidification Rate (PDF 83.2 KB) [file 11357_2025_2033_MOESM7_ESM.pdf]
