# Supplementary material for: A multi-omics analysis of human fibroblasts overexpressing an Alu transposon reveals widespread disruptions in aging-associated pathways
Source: GeroScience. 2025 Dec 11;48(3):3375–402. doi: 10.1007/s11357-025-02033-6 (PMC13356197; doi:10.1007/s11357-025-02033-6)
Supplement: Supplementary file 3 — Supplementary Fig. S3 AluJb-induced transcriptomic changes partially mirror AluS-induced changes. (a) A diagram illustrating how AluJb-induced and previously published AluS-induced transcriptomic changes were compared. (b) Mosaic plots showing the fraction of AluS-induced genes found among significant (FDR < 0.05) and non-significant AluJb-induced genes. Statistical significance of frequency differences was assessed with Fisher’s exact test, and p < 0.05 was considered significant. (c) GSEA analysis with gene sets for AluS upregulated and downregulated genes following AluJb overexpression. Gene sets with FDR < 0.05 were considered significant. GSEA: Gene Set Enrichment Analysis, FDR: False Discovery Rate, NES: Normalized Enrichment Score (PDF 193 KB) [file 11357_2025_2033_MOESM3_ESM.pdf]

Fig. S3

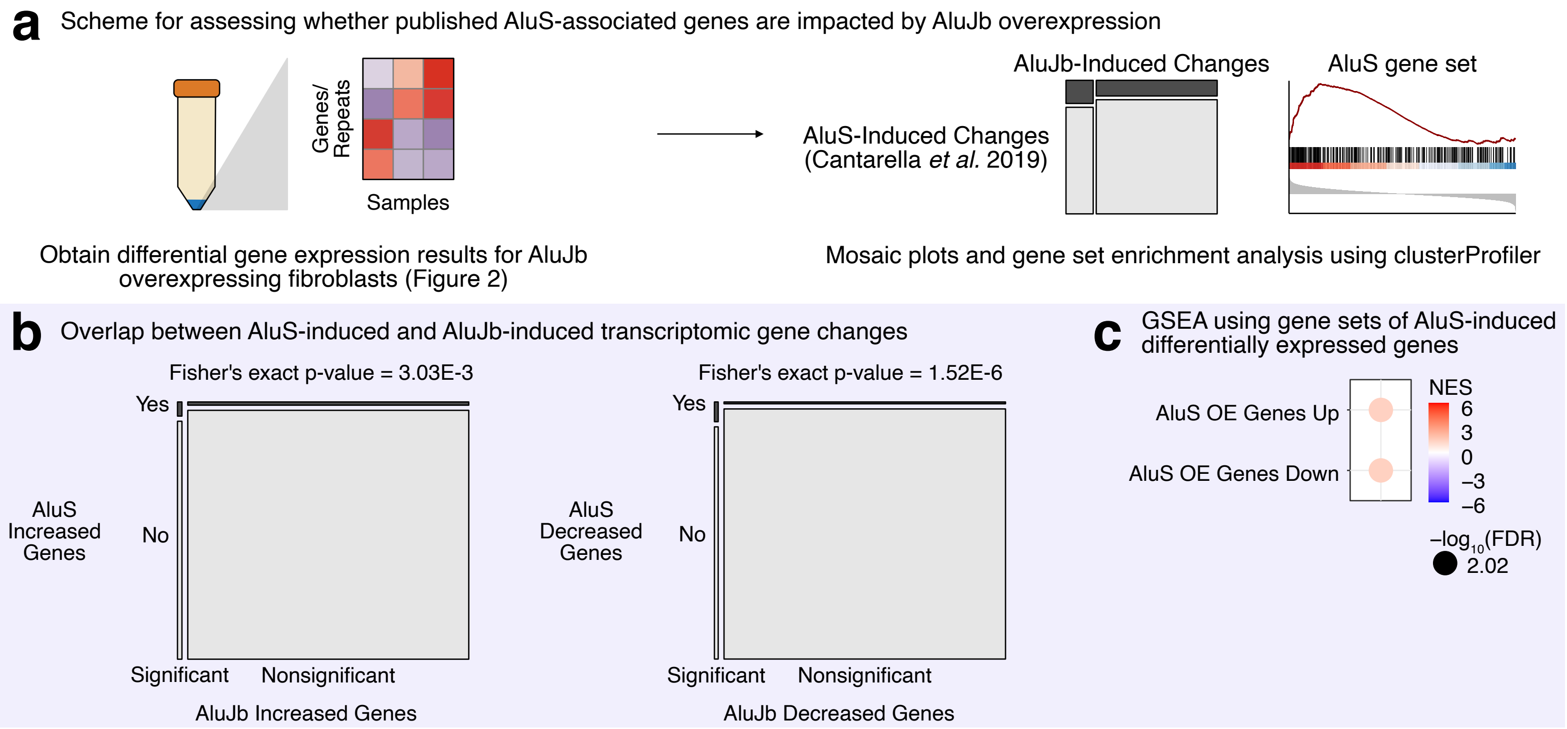
a
Scheme for assessing whether published AluS-associated genes are impacted by AluJb overexpression
Genes/
Repeats
Samples
AluJb-Induced Changes
AluS gene set
AluS-Induced Changes
(Cantarella et al. 2019)
Obtain differential gene expression results for AluJb overexpressing fibroblasts (Figure 2)
Mosaic plots and gene set enrichment analysis using clusterProfiler
b
Overlap between AluS-induced and AluJb-induced transcriptomic gene changes
Fisher's exact p-value = 3.03E-3
Yes
No
AluS Increased Genes
Significant
Nonsignificant
AluJb Increased Genes
Fisher's exact p-value = 1.52E-6
Yes
No
AluS Decreased Genes
Significant
Nonsignificant
AluJb Decreased Genes
c
GSEA using gene sets of AluS-induced differentially expressed genes
AluS OE Genes Up
AluS OE Genes Down
NES
6
3
0
−3
−6
−log10(FDR)
2.02
