# Supplementary material for: A multi-omics analysis of human fibroblasts overexpressing an Alu transposon reveals widespread disruptions in aging-associated pathways
Source: GeroScience. 2025 Dec 11;48(3):3375–402. doi: 10.1007/s11357-025-02033-6 (PMC13356197; doi:10.1007/s11357-025-02033-6)
Supplement: Supplementary file 4 — Supplementary Fig. S4 AluJb-induced transcriptomic changes are partially induced by aging. (a) A diagram illustrating how AluJb-induced and aging-induced transcriptomic changes were compared. Mosaic plots showing the fraction of AluJb-induced (b) genes and (d) repeats found among significant (FDR < 0.05) and non-significant primary fibroblast aging-induced genes and repeats. Statistical significance of frequency differences was assessed with Fisher’s exact test, and p < 0.05 was considered significant. GSEA analysis with gene sets for AluJb upregulated and downregulated (c) genes and (e) repeats in human aging primary fibroblasts. Gene sets with FDR < 0.05 were considered significant. GSEA: Gene Set Enrichment Analysis, FDR: False Discovery Rate, NES: Normalized Enrichment Score (PDF 198 KB) [file 11357_2025_2033_MOESM4_ESM.pdf]

Fig. S4

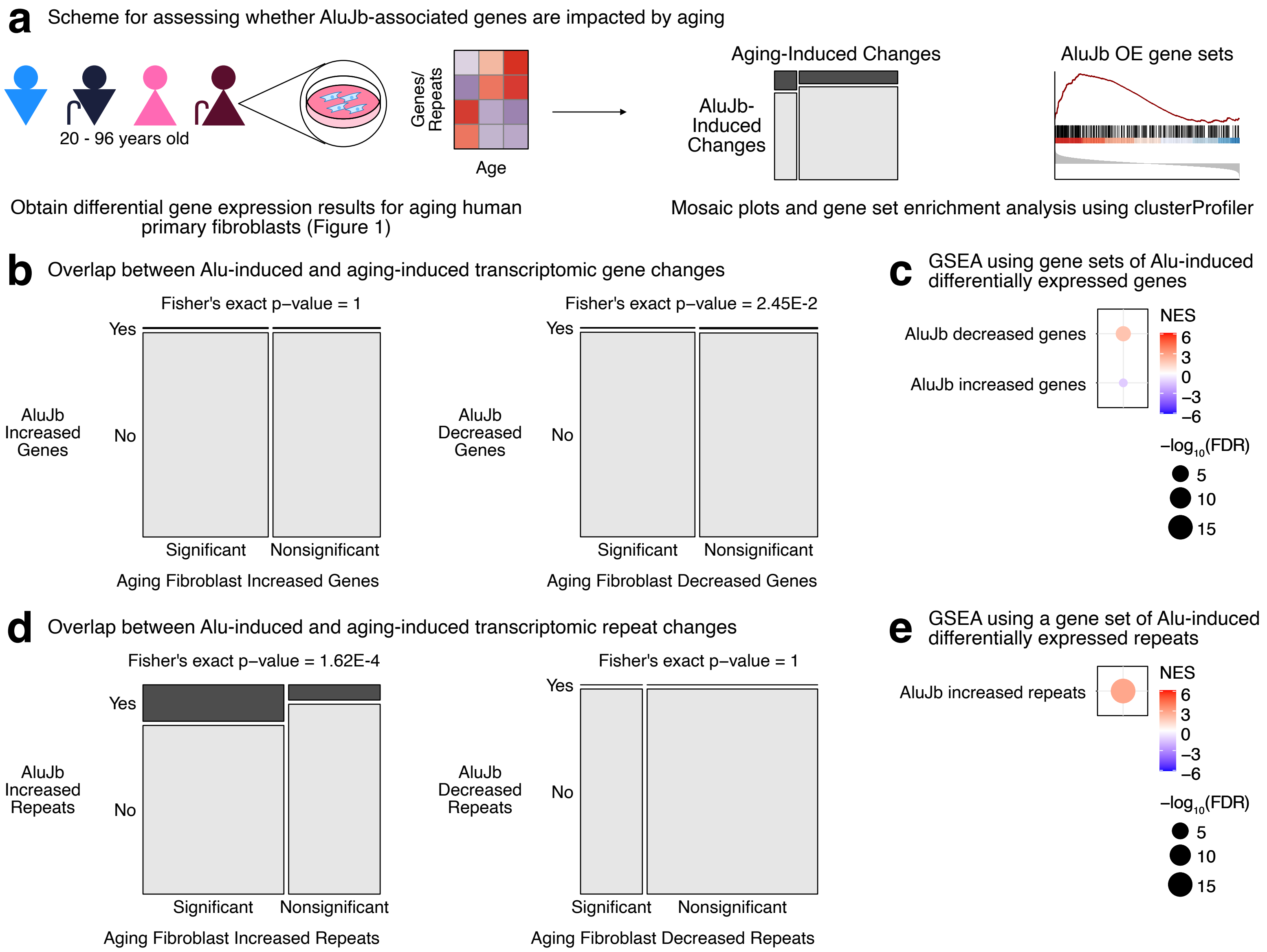
a
Scheme for assessing whether AluJb-associated genes are impacted by aging
20 - 96 years old
Genes/ Repeats
Age
Obtain differential gene expression results for aging human primary fibroblasts (Figure 1)
Aging-Induced Changes
AluJb-Induced Changes
AluJb OE gene sets
Mosaic plots and gene set enrichment analysis using clusterProfiler
b
Overlap between Alu-induced and aging-induced transcriptomic gene changes
Fisher's exact p–value = 1
Yes
No
AluJb Increased Genes
Significant
Nonsignificant
Aging Fibroblast Increased Genes
Fisher's exact p–value = 2.45E-2
Yes
No
AluJb Decreased Genes
Significant
Nonsignificant
Aging Fibroblast Decreased Genes
c
GSEA using gene sets of Alu-induced differentially expressed genes
AluJb decreased genes
AluJb increased genes
NES
6
3
0
–3
–6
–log10(FDR)
5
10
15
d
Overlap between Alu-induced and aging-induced transcriptomic repeat changes
Fisher's exact p–value = 1.62E-4
Yes
No
AluJb Increased Repeats
Significant
Nonsignificant
Aging Fibroblast Increased Repeats
Fisher's exact p–value = 1
Yes
No
AluJb Decreased Repeats
Significant
Nonsignificant
Aging Fibroblast Decreased Repeats
e
GSEA using a gene set of Alu-induced differentially expressed repeats
AluJb increased repeats
NES
6
3
0
–3
–6
–log10(FDR)
5
10
15
