# Supplementary material for: A multi-omics analysis of human fibroblasts overexpressing an Alu transposon reveals widespread disruptions in aging-associated pathways
Source: GeroScience. 2025 Dec 11;48(3):3375–402. doi: 10.1007/s11357-025-02033-6 (PMC13356197; doi:10.1007/s11357-025-02033-6)
Supplement: Supplementary file 5 — Supplementary Fig. S5 AluJb induces similar widespread changes in proliferating and serum-deprived fibroblasts. (a) A diagram illustrating how the transcriptomic impact of AluJb overexpression in proliferating IMR-90 fibroblasts was assessed. (b) Plasmid-specific AluJb overexpression was assessed by endpoint RT-PCR of empty vector and AluJb overexpressing IMR-90 fibroblasts using primers targeting the 3’ AluJb-plasmid backbone junction. PCR reactions were carried out with (RT+) or without (RT-) reverse transcription in N = 4 samples per group, and all of these were used for transcriptomic profiling. Multidimensional scaling (MDS) analysis of the (c) gene and (d) repetitive element transcriptomes across samples. (e) Quantification of the percent of total reads mapping to repetitive elements. Statistical significance was assessed with a Wilcoxon rank sum test. (f) Gene expression heatmaps for significant (FDR < 0.05) differentially expressed genes and repeat subfamilies. The top 5 significant (FDR < 0.05) and commonly regulated (g) GO Biological Process and (h) Reactome pathway gene sets in serum-deprived fibroblasts and proliferating fibroblasts in standard media. (i) Hallmark interferon alpha and gamma gene set regulation in serum-deprived, quiescent cells versus proliferating cells cultured in standard rich media. The grey overlay indicates FDR > 10% (no trends for significance). Fisher’s method was used to combine p-values from GSEA analyses in each media condition, and pathways were ranked on their meta-analysis p-value. RT: Reverse Transcription, GSEA: Gene Set Enrichment Analysis, FDR: False Discovery Rate, NES: Normalized Enrichment Score (PDF 1.34 MB) [file 11357_2025_2033_MOESM5_ESM.pdf]

Fig. S5

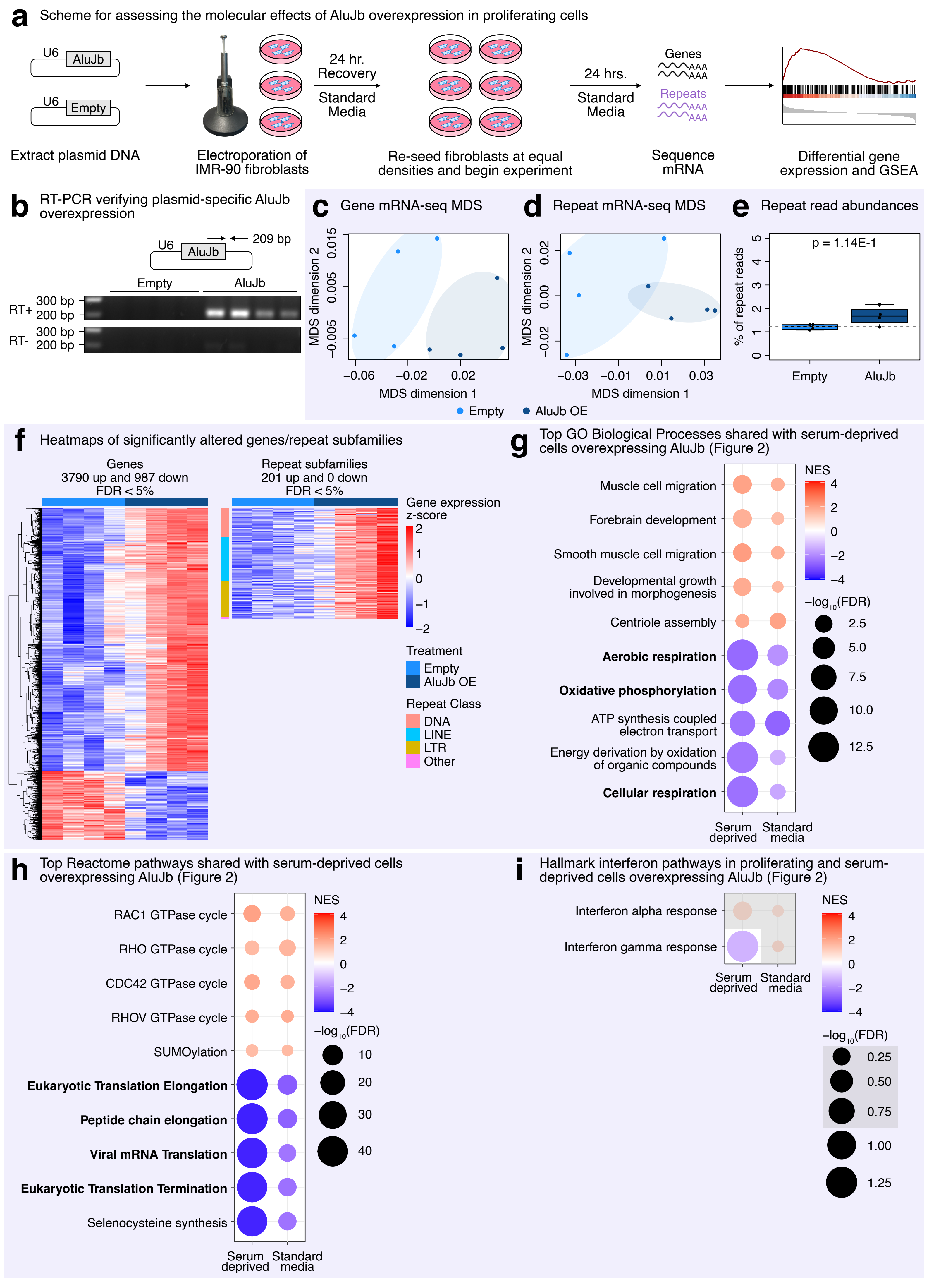

a
Scheme for assessing the molecular effects of AluJb overexpression in proliferating cells
U6
AluJb
U6
Empty
Extract plasmid DNA
Electroporation of IMR-90 fibroblasts
24 hr. Recovery
Standard Media
Re-seed fibroblasts at equal densities and begin experiment
24 hrs.
Standard Media
Genes
AAA
Repeats
AAA
Sequence mRNA
Differential gene expression and GSEA
b
RT-PCR verifying plasmid-specific AluJb overexpression
209 bp
Empty
AluJb
RT+
RT-
300 bp
200 bp
c
Gene mRNA-seq MDS
MDS dimension 1
MDS dimension 2
d
Repeat mRNA-seq MDS
e
Repeat read abundances
p = 1.14E-1
% of repeat reads
Empty
AluJb
Empty
AluJb OE
f
Heatmaps of significantly altered genes/repeat subfamilies
Genes
3790 up and 987 down
FDR < 5%
Repeat subfamilies
201 up and 0 down
FDR < 5%
Gene expression z-score
Treatment
Empty
AluJb OE
Repeat Class
DNA
LINE
LTR
Other
g
Top GO Biological Processes shared with serum-deprived cells overexpressing AluJb (Figure 2)
Muscle cell migration
Forebrain development
Smooth muscle cell migration
Developmental growth involved in morphogenesis
Centriole assembly
Aerobic respiration
Oxidative phosphorylation
ATP synthesis coupled electron transport
Energy derivation by oxidation of organic compounds
Cellular respiration
NES
−log10(FDR)
2.5
5.0
7.5
10.0
12.5
Serum deprived
Standard media
h
Top Reactome pathways shared with serum-deprived cells overexpressing AluJb (Figure 2)
RAC1 GTPase cycle
RHO GTPase cycle
CDC42 GTPase cycle
RHOV GTPase cycle
SUMOylation
Eukaryotic Translation Elongation
Peptide chain elongation
Viral mRNA Translation
Eukaryotic Translation Termination
Selenocysteine synthesis
NES
−log10(FDR)
10
20
30
40
Serum deprived
Standard media
i
Hallmark interferon pathways in proliferating and serum-deprived cells overexpressing AluJb (Figure 2)
Interferon alpha response
Interferon gamma response
NES
−log10(FDR)
0.25
0.50
0.75
1.00
1.25
Serum deprived
Standard media
