# Supplementary material for: A multi-omics analysis of human fibroblasts overexpressing an Alu transposon reveals widespread disruptions in aging-associated pathways
Source: GeroScience. 2025 Dec 11;48(3):3375–402. doi: 10.1007/s11357-025-02033-6 (PMC13356197; doi:10.1007/s11357-025-02033-6)
Supplement: Supplementary file 6 — Supplementary Fig. S6 Genes and proteins differentially regulated across “omic” analyses. The abundances of four significantly altered genes—POSTN, NES, DCN, and DBI—and their proteins in the (a) transcriptome, (b) cell proteome, and (c) secretome. FDR: False Discovery Rate, Q: Q-value (PDF 39.7 KB) [file 11357_2025_2033_MOESM6_ESM.pdf]

Fig. S6

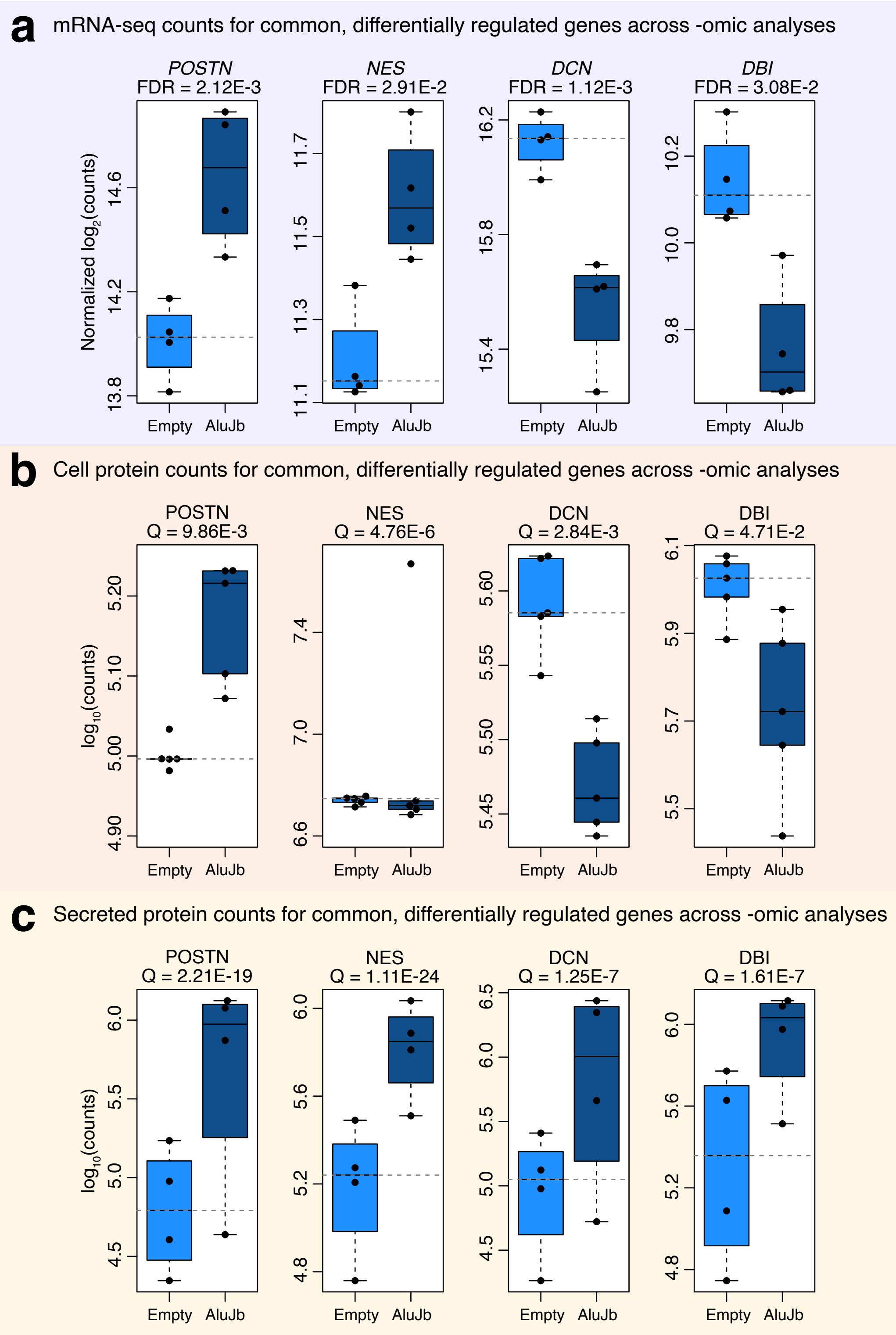
a
mRNA-seq counts for common, differentially regulated genes across -omic analyses
POSTN
FDR = 2.12E-3
NES
FDR = 2.91E-2
DCN
FDR = 1.12E-3
DBI
FDR = 3.08E-2
Normalized log2(counts)
Empty
AluJb
b
Cell protein counts for common, differentially regulated genes across -omic analyses
POSTN
Q = 9.86E-3
NES
Q = 4.76E-6
DCN
Q = 2.84E-3
DBI
Q = 4.71E-2
log10(counts)
Empty
AluJb
c
Secreted protein counts for common, differentially regulated genes across -omic analyses
POSTN
Q = 2.21E-19
NES
Q = 1.11E-24
DCN
Q = 1.25E-7
DBI
Q = 1.61E-7
log10(counts)
Empty
AluJb
